# Clinical and hormonal characteristics and growth data of 45,X/46,XY mosaicism in 38 Chinese patients

**DOI:** 10.3389/fped.2023.1135776

**Published:** 2023-04-19

**Authors:** Jiaoru Yang, Yan Li, Pin Li

**Affiliations:** Department of Endocrinology, Shanghai Children’s Hospital, Children’s Hospital Affiliated to Shanghai Jiao Tong University School of Medicine, Shanghai, China

**Keywords:** height, weight, BMI, sex hormones, correlation, 45,X/46,XY mosaicism

## Abstract

**Backgrounds:**

45,X/46,XY mosaicism is the most common type of sex chromosomal abnormality in disorders of sex development (DSD). We investigated the clinical manifestations, serum sex hormone levels and growth data of 38 45,X/46,XY mosaicism patients, which provides better insight into this disease.

**Methods:**

We prospectively evaluated 38 patients who were diagnosed with 45,X/46,XY mosaicism at the Department of Endocrinology of Shanghai Children's Hospital from 2010 to 2020. We analyzed clinical data from the patients, including hormone levels, height, weight, body mass index (BMI) and gonadal pathology results.

**Results:**

Among the 38 cases of 45,X/46,XY mosaicism, 18 cases showed a female external genitalia phenotype (the female group) with an external masculinization score (EMS) of 1 (0–3) [median (range)], and 20 cases showed a male external genitalia phenotype (the male group) with an EMS of 7.63 (3–11) [median (range)]. The age at diagnosis ranged from 0.7 to 16.1 years. Under 2 years of age, the standard deviation scores of height (HtSDS) were in the normal range and then they gradually decreased. The inhibin B (INHB), anti-Mullerian hormone (AMH), and testosterone (T) levels after human chorionic gonadotropin (HCG) stimulation and the T:DHT ratio in the male group were significantly higher than those in the female group (*P *< 0.001). The basal luteinizing hormone (LH), basal follicle-stimulating hormone (FSH), peak LH and peak FSH in females were significantly higher than those in males (*P *< 0.05). Their height showed a positive correlation with T levels after HCG stimulation (*r *= 0.636, *P* < 0.01), T:DHT ratio (*r *= 0.724, *P* < 0.01), growth hormone (GH) (*r *= 0.827, *P* < 0.05), and insulin-like growth factor 1 (IGF-1) (*r* = 0.067, *P* > 0.05) and a negative correlation with gonadal pathology in ovarian tissue (*r* = −0.663, *P* < 0.05) and the number of chimaeric XY cells (*r* = −0.533, *P* < 0.05).

**Conclusions:**

Patients with 45,X/46,XY mosaicism have specific growth patterns. Their HtSDS was in the normal range during 0–2 years of age and then they began to show a short stature after 2 years of age. The probability of short stature in females was higher than that in males. WtSDS were all in the normal range, but below the median. BMISDS was in the normal range, and there was no evidence of obesity. The gonads in the male group retained a certain androgen secretion function, while the gonadal damage is more severe in the female group.

## Background

Disorders/differences of sex development (DSD) are congenital disorders of abnormal development of the internal and external genitalia, and these developmental disorders are usually caused by abnormal gonadal determination and differentiation (1). Among the various DSD conditions, there are sex chromosome DSDs due to numerical or structural abnormalities of the sex chromosomes (1). 45,X/46,XY mosaicism is a rare sex chromosome DSD associated with a broad spectrum of clinical phenotypes, from Turner females to phenotypically normal males with varying degrees of genital ambiguity (2). The sex of rearing may be male or female based on the appearance of the genitalia at birth (2). At present, there are few reports on Chinese 45,X/46,XY mosaicism. We retrospectively studied the clinical manifestations, hormone levels, and growth data of 38 patients with 45,X/46,XY DSD, which provides better insight into this disease.

## Methods

### Subjects

Informed consent from family members and patients was obtained before the study and approved by the Hospital Ethics Committee (2021R092-E01). In this study, 38 patients diagnosed with 45,X/46,XY mosaicism at the Department of Endocrinology of Shanghai Children's Hospital from 2010 to 2020 were selected as the research subjects. These patients were divided into a male phenotype group (20 cases) and a female phenotype group (18 cases) according to sex of rearing. Comparisons of height, weight and BMI were made against population norms.

### Karyotyping

Blood sample (0.3–0.5 ml, heparin anticoagulation) was added into cell culture medium (Dubai Biomedical Co. Ltd.,Shanghai, China). Thirty to 100 mitoses were examined to determine the percentage of cell line mosaicism. All karyotypes were evaluated by an experienced clinical geneticist, and according ISCN 2016. We use the ratio of the number of 46,XY mosaicism or 45,X mosaicism over the total number of mitoses to reflect the relative presence of the number of Y-chromosomes or 45,X monosomy.

### External genital phenotype evaluation

We evaluated the external genital phenotypes according to the External Masculinization Score (EMS) (3), when they first visit in endocrinology, and all before surgery. The measurement method of the penis is manual measurement (4), measuring the length of the penis stretched while the subject is in a supine position. The measurer places the end of a ruler against the pubic symphysis, lifts the head of the penis with the thumb and index finger and gradually applies it along the length of the penis. Traction stretches the penis until the penis is stretched to its longest length. The length from the end of the ruler to the apex of the glans penis is the stretched length (the length of the foreskin is not counted). The length of the penis was compared with the data of normal Chinese children. The location of the testes was determined by physical examination and ultrasonography.

### Hormonal analysis

To evaluate the hypothalamic-pituitary-gonadal (HPG) axis and testicular function, all patients underwent gonadotropin-releasing hormone (GnRH) stimulation and human chorionic gonadotropin (HCG) stimulation. Sex hormones, including basal testosterone (T), basal dihydrotestosterone (DHT), oestradiol (E2), basal luteinizing hormone (LH), basal follicle-stimulating hormone (FSH), anti-Mullerian hormone (AMH), inhibin B (INHB), sex hormone binding globulin (SHBG), insulin-like growth factor-1 (IGF-1), insulin-like growth factor-binding protein-3 (IFGBP-3), peak LH and peak FSH after gonadotropin-releasing hormone (GnRH) stimulation and T and DHT after human HCG stimulation, were detected. Detection methods: E2, T and DHT were tested by ELISA and measured with a USA Polar ELx800 microplate reader. Serum LH and FSH concentrations were tested with LH and FSH detection kits (Beckman Coulter) and measured with an automatic immunoluminescence analyser (UnicelDxI 800). Serum AMH and INHB were detected with solid-phase sandwich enzyme-linked immunosorbent assays (ELISAs) purchased from Guangzhou Kangrun Biotechnology Co., Ltd. IGF-1 and IGFBP-3 were detected by Siemens Medical Diagnostics Chemiluminescence Analyser IMMULITE 2000.

### Statistical analysis

SPSS 25.0 software was used for the statistical analyses, and GraphPad Prism 9.0 was used for graphing. All detection indicators were tested for normality. The normally distributed data are expressed as the mean ± SD (x ± s), and the data with a nonnormal distribution are expressed as the median (upper quartile to lower quartile). The nonparametric Mann‒Whitney rank-sum test was used to compare two groups with a nonnormal distribution. Bivariate correlation analysis was performed using Pearson's method, and the correlation coefficient was denoted by *r*. Then, the correlations between height, weight and each index were analysed by multiple stepwise regression. *P *< 0.05 was considered statistically significant.

### Growth curve plotting

The calculations for the growth curves were performed using LMS-Chartmaker Pro software, and the curves were drawn using Excel 2019. The internationally well accepted method (λ-median coefficient of variation, LMS) for generating standard curves was adopted to calculate the M, S, and L (after converting the data into a normal distribution, using Box‒Cox transformation) (5), which described the growth index in each age group.

## Results

### External genitalia phenotype

Thirty-eight patients were scored for external genital virilization according to the EMS. The results showed that the EMS of patients raised as females was 1 (0–3) [median (range)] and that of the males was 7.63 (3–11) [median (range)] (*P *= 0.0001 < 0.001) (Table 1).

**Table 1:** EMS of 45,X/46,XY mosaicism patients.

Patient no.	Diagnosis		Sex	Karyotype	At last follow-up			EMS					
	Age (year)	Reason for referral			Age (month)	Height (cm)	Weight (kg)	Scrotal fusion	Micropenis	Urethral meatus	Right gonad	Left gonad	Total EMS
1	At birth	Abnormal genitals	M	45,X(15)/46,XY(15)	11	71	9.4	3	3	2	1	0	9
2	At birth	Abnormal genitals	M	45,X(15)/46,XY(15)	37	83.5	10.5	0	0	1	0.5	0.5	2
3	At birth	Abnormal genitals	M	45,X(6)/46,XY(24)	8	72	10	3	0	0	1.5	0	4.5
4	At birth	Abnormal genitals	M	45,X(2)/46,XY(18)	34	93	11.75	0	3	1	1	1	6
5	At birth	Abnormal genitals	M	45,X(45)/46,XY(15)	19	76.5	10	0	3	1	1	0.5	5.5
6	At birth	Abnormal genitals	M	45,X(10)/46,XY(25)	11	73	9.4	3	0	0	0.5	0.5	4
7	At birth	Abnormal genitals	M	45,X(16)/46,XY(34)	12	75	10.6	0	0	0	1.5	0.5	2
8	At birth	Abnormal genitals	M	45,X(15)/46,XY(35)	11	76	11.4	0	0	1	0.5	0	1.5
9	At birth	Abnormal genitals	M	45,X(56)/46,XY(6)	38	93	12.8	3	3	0	1	1	8
10	9	Growth retardation	M	45,X(8)/46,XY(91)	111	113	18.5	3	3	2	1.5	1.5	11
11	At birth	Abnormal genitals	M	45,X(23)/46,XY(77)	18	76	10	3	3	1	1	1	9
12	At birth	Abnormal genitals	M	45,X(7)/46,XY(20)	11	71	9	3	3	2	0.5	0.5	9
13	At birth	Abnormal genitals	M	45,X(40)/46,XY(10)	15	75	10	0	0	0	1	1.5	2.5
14	At birth	Abnormal genitals	M	45,X(38)/46,XY(12)	14	72	9.5	0	0	1	1	0.5	2.5
15	At birth	Abnormal genitals	M	45,X(16)/46,XY(30)	25	88	10.9	0	0	1	0.5	0.5	2
16	At birth	Abnormal genitals	M	45,X(39)/46,XY(44)	13	76	9.8	3	0	0	1	1	5
17	At birth	Abnormal genitals	M	45,X(17)/46,XY(83)	69	98	14.6	3	0	0	1	1.5	5.5
18	At birth	Abnormal genitals	M	45,X(7)/46,XY(13)	10	70	8.7	0	3	2	0.5	1	6.5
19	At birth	Abnormal genitals	M	45,X(19)/46,XY(81)	37	86	12.4	0	3	2	0	0.5	5.5
20	At birth	Abnormal genitals	M	45,X(9)/46,XY(41)	13	71	9	3	0	2	1	0.5	6.5
21	11	Growth retardation	F	45,X(5)/46,XY(17)	136	138	43	0	0	0	0	0	0
22	8	Growth retardation	F	45,X(31)/46,XY(29)	98	115	23.05	0	0	1	0	0	1
23	13	Growth retardation	F	45,X(5)/46,XY(55)	164	144	39.3	0	0	0	0	0	0
24	8	Growth retardation	F	45,X(22)/46,XY(8)	101	118.3	23	0	0	0	0	0	0
25	At birth	Abnormal genitals	F	45,X(14)/46,XY(16)	12	78	10	0	0	0	0	0	0
26	At birth	Abnormal genitals	F	45,X(15)/46,XY(35)	12	72	8.1	0	3	0	0	0	3
27	15	delayed puberty	F	45,X(19)/46,XY(31)	187	149	46.8	0	0	0	0.5	0.5	1
28	At birth	Abnormal genitals	F	45,X(33)/46,XY(17)	17	78	9.4	0	0	0	1	1	2
29	11	Growth retardation	F	45,X(74)/46,XY(26)	141	120	26.51	0	0	0	0	0	0
30	At birth	Abnormal genitals	F	45,X(30)/46,XY(70)	45	93	12.6	0	0	0	0	0	0
31	4	Growth retardation	F	45,X(11)/46,XY(89)	28	87	11.5	0	0	0	0	0	0
32	4	Growth retardation	F	45,X(60)/46,XY(40)	53	98	16.5	0	3	0	0	0	3
33	3	Growth retardation	F	45,X(72)/46,XY(28)	74	99	14.35	0	0	2	0.5	0.5	3
34	At birth	Abnormal genitals	F	45,X(17)/46,XY(83)	76	109	18.3	0	0	0	0.5	0.5	1
35	8	Growth retardation	F	45,X(34)/46,XY(16)	120	121	26.9	0	0	0	0	0	0
36	16	delayed puberty	F	45,X(42)/46,XY(18)	193	148	39.5	0	0	0	0	0	0
37	11	Growth retardation	F	45,X(15)/46,XY(25)	178	140	44	0	0	1	0	0	1
38	7	Growth retardation	F	45,X(6)/46,XY(24)	122	131	36.1	0	0	1	0	0	1

M, Male; F, female; EMS, External Masculinization Score;Scores (yes/no or gradient): scrotal fusion (3/0), micropenis (0/3), urethral meatus (normal, 3; glandular, 2; penile, 1; perineal, 0), right and left gonad (scrotal, 1.5; inguinal, 1; abdominal, 0.5; absent, 0).

### Growth pattern

**Figure 1:**
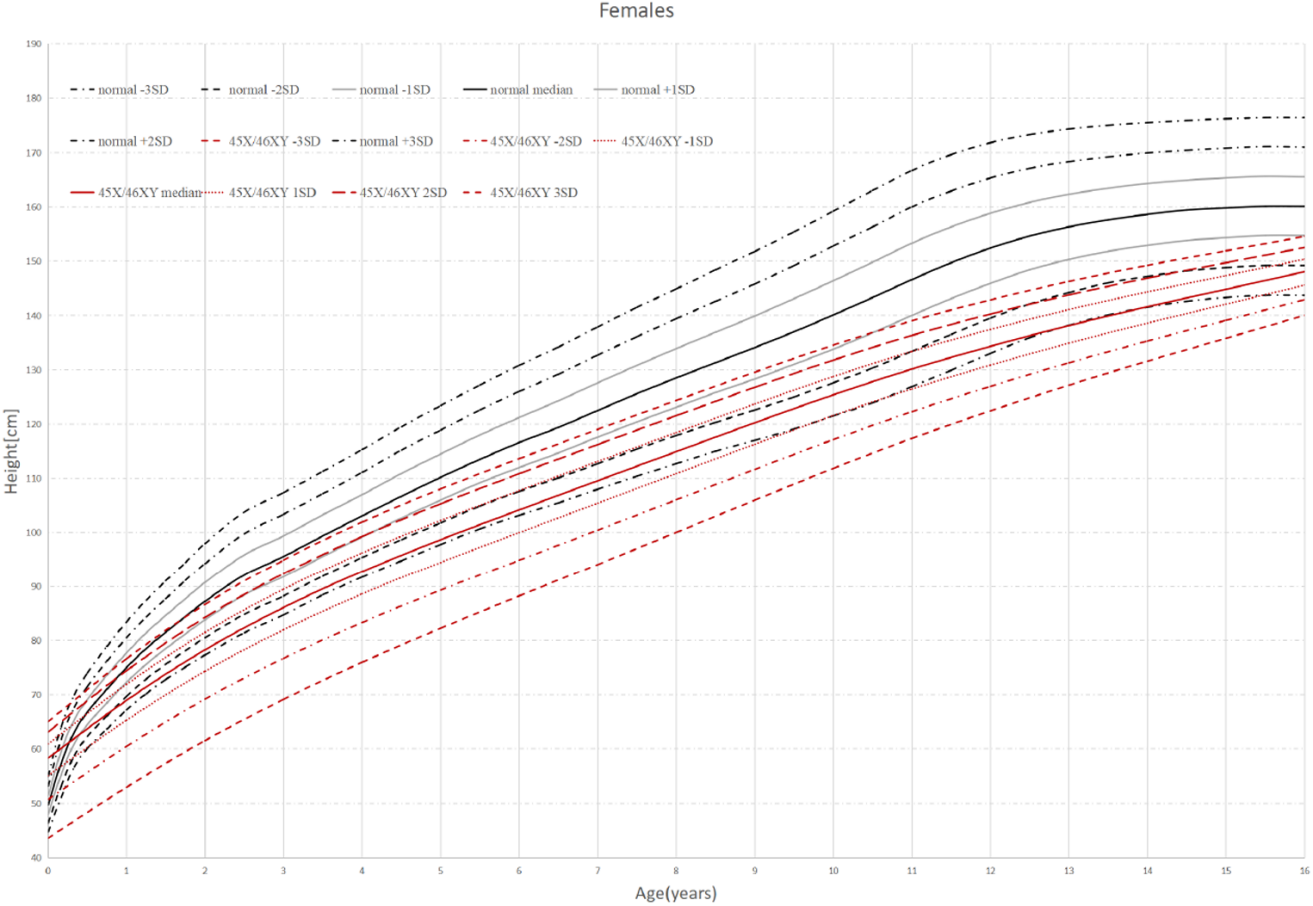
Standard deviation height curves of children with 45,X/46,XY mosaicism and normal Chinese girls.

**Figure 2:**
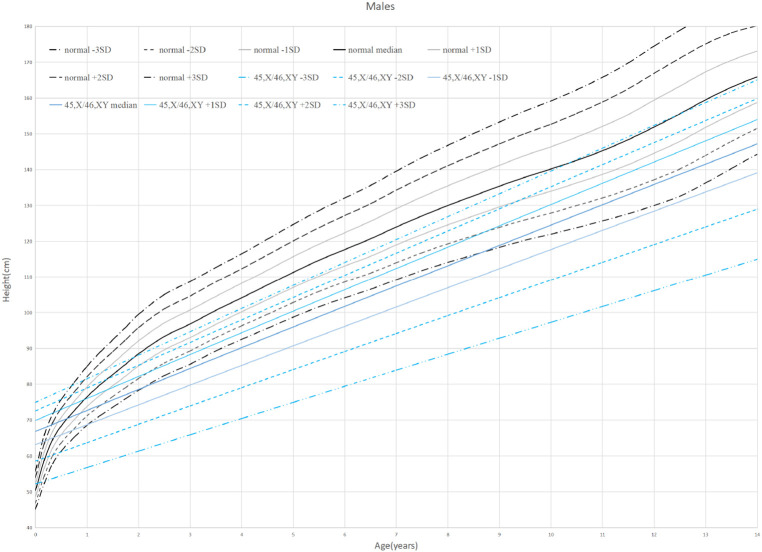
Standard deviation height curves of children with 45,X/46,XY mosaicism and normal Chinese boys.

Thirty-eight patients were divided into 6 groups according to age: 0–1 years old, 1–2 years old, 2–6 years old, 6–9 years old, 9–13 years old, and older than 13 years old. When the children were under 2 years old, their HtSDS was in the normal range (−0.9 ± 1.16, −1.79 ± 0.85) and then it gradually decreased. Their height began to appear short and was the lowest at the age of 9–13 (−3.17 ± 1.71) (*P *= 0.014 < 0.05). The lowest and highest weight standard deviation scores were at 6–9 years old and 0–1 years old (−1.94 ± 0.83, −0.51 ± 0.99) (*P *= 0.026 < 0.05). The lowest and highest BMIs were in the 2- to 6-year-old group and the older than 13-year-old group (15.16 ± 1.16, 20.13 ± 2.01) (*P* < 0.0001 < 0.001). The above results show that the growth of 45,X/46,XY mosaicism children appeared to decelerate around 2 years of age, and after that point, their height was significantly lower than that of normal children (Figures 1, 2). Below −2 SD children with this disease were underweight but within the range of normal children with a normal BMI (Table 2).

**Table 2:** Growth of 45,X/46,XY mosaicism in different age groups.

Age	*N*	HtSDS△	WtSDS△	BMISDS△
0–1 year	10	−0.90 ± 1.16	−0.51 ± 0.99	0.30 ± 0.63
1–2 years	6	−1.79 ± 0.85	−0.9 ± 0.33	0.07 ± 0.56
2–6 years	9	−2.14 ± 1.38	−1.90 ± 0.86	−0.38 ± 1.19
6–9 years	4	−2.91 ± 0.59	−1.94 ± 0.83	−0.16 ± 1.81
9–13 years	5	−3.17 ± 1.71	−1.14 ± 1.75	0.77 ± 1.21
>13 years	4	−2.9 ± 1.34	−1.14 ± 0.63	0.29 ± 0.77
F		3.385	2.487	0.978
*P*		0.014*	0.052	0.446

*N*, number; HtSDS, height standard deviation scores; WtSDS,weight standard deviation scores; BMISDS, body mass index standard deviation score; *P < 0.05. △ indicates that according to the SK normality test, it follows a normal distribution.

Thirty-eight patients were divided into female (18 cases) and male (20 cases) groups according to the phenotype of their external genitalia. The HtSDS of females was −2.29 ± 1.42 and that of males was −1.85 ± 1.43, and the probability of short stature in females (67%) was higher than that in males (45%). The WtSDS of females and males was −1.26 ± 1.036 and −1.14 ± 1.14, respectively, both lower than the median but still within normal levels. The BMISDS of females was higher than that of males (*P *= 0.029 < 0.05) (Table 3). The above results suggest that 45,X/46,XY mosaicism children raised as girls have obvious androgen deficiency, which seriously affects their height growth and causes more severe short stature.

**Table 3:** 45,X/46,XY mosaicism female and male group height, weight, BMI levels.

	*N*	HtSDS△	WtSDS△	BMISDS△
Females	18	−2.29 ± 1.42	−1.26 ± 1.036	0.43 ± 1.17
Males	20	−1.85 ± 1.43	−1.14 ± 1.14	−0.8 ± 1.08
F		0.91	0.111	5.521
*P*		0.347	0.741	0.029*

*N*, number; HtSDS, height standard deviation scores; WtSDS,weight standard deviation scores; BMISDS, body mass index standard deviation score;*P < 0.05. △ indicates that according to the SK normality test the data follow a normal distribution.

### Hormones

**Figure 3:**
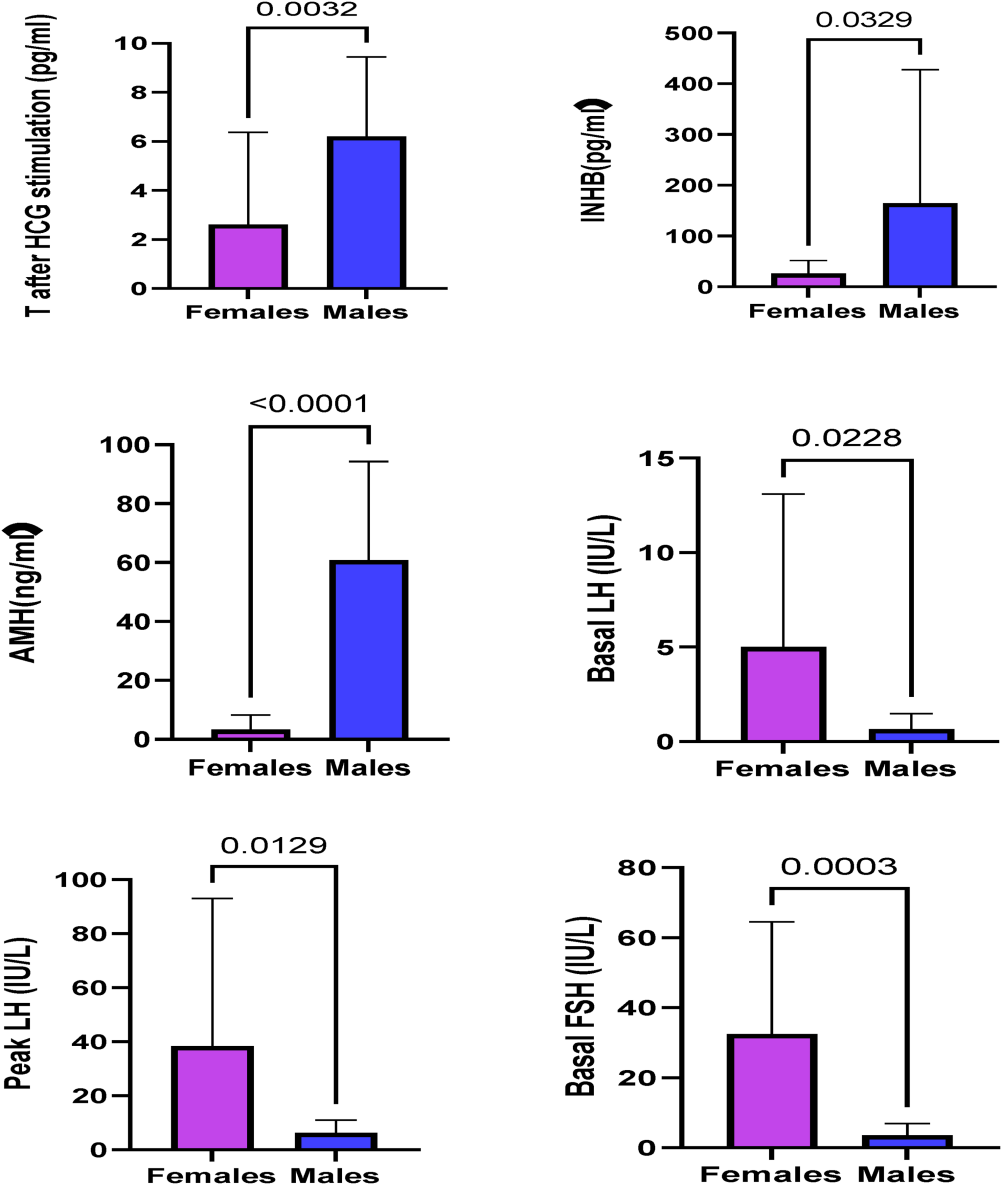
Hormones showing significant differences in the sex of 45,X/46,XY patients.

The INHB, AMH, and T levels after HCG stimulation and the T:DHT ratio in the male group were significantly higher than those in the female group (*P *< 0.001), and the basal LH, basal FSH, peak LH and peak FSH in the female group were significantly higher than those in the male group (*P *< 0.05) (Table 4). This suggests that the male group has a certain level of testicular function and can secrete androgens after HCG stimulation, while the female group has the risk of manifesting hypergonadotropic hypogonadism with insufficient testosterone secretion (Figure 3).

**Table 4:** 45,X/46,XY mosaicism male and female hormone levels.

	Females (*N* = 18)	Males (*N* = 20)	Z value	*P*-value
Basal T (nmol/L)^a^	0.35 (0.35–0.665)	0.35 (0.35–0.61)	−0.657	0.511
Basal DHT (pg/ml)^a^	102.3 (28.64–224.53)	73.86 (29.92–167.63)	−0.38	0.704
DHEAS (umol/L)^a^	0.9 (0.2–2.4)	0.1 (0.05–0.38)	−3.472	0.001**
INHB (pg/mL)^a^	12.85 (9.31–42.48)	89.43 (63.07–181.82)	−4.619	0.000***
AMH (ng/mL)^a^	1.26 (0.47–4.19)	54.94 (36.6–73)	−5.263	0.0008**
T after HCG stimulation (nmol/L)^a^	1.2 (0.645–2.38)	5.44 (3.195–9.7425)	−3.626	0.000***
DHT after HCG stimulation (pg/ml)^a^	156.7 (61.93–230.3)	111.59 (55.42–221.53)	−0.643	0.52
T:DHT ratio^a^	2.22 (1.01–8.4)	13.87 (8.58–28.68)	−3.625	0.000***
Basal LH (IU/L)^a^	0.87 (0.27–9.04)	0.39 (0.2–0.67)	−2.016	0.044*
Basal FSH (IU/L)^a^	17.72 (6.35–53.87)	2.96 (1.79–4.23)	−4.444	0.000***
Peak LH (IU/L)^a^	16.54 (7.14–37.15)	5.55 (3.65–7.55)	−3.459	0.001**
Peak FSH (IU/L)^a^	106.75 (43.51–119.95)	18.61 (14.84–24.27)	−4.785	0.000***

T, Testosterone; DHT, Dihydrotestosterone; INHB, Inhibin B; AMH, Anti-Mullerian hormone; DHEAS, Dehydroepiandrosterone sulfate; LH, Luteinizing hormone; FSH, Follicle stimulating hormone; HCG, Human chorionic gonadotropin.

^a^ Indicates that the SK normality test does not obey the normal distribution.

* *P* < 0.05, **P < 0.01, ***P < 0.001.

### Correlation analysis

The correlation analysis results of height, weight and BMI of the 45,X/46,XY children are shown in Tables 5, 6. The 45,X/46,XY children's height showed a positive correlation with T (after HCG stimulation) (*r *= 0.636, *P* < 0.01), the T:DHT ratio (*r *= 0.724, *P* < 0.01), GH (*r *= 0.827, *P* < 0.05), and IGF-1 (*r *= 0.067, *P* > 0.05) and a negative correlation with gonadal pathology in ovarian tissue (*r* = −0.663, *P* < 0.05) and the number of XY chimaeras (*r* = −0.533, *P* < 0.05). WtSDS was positively correlated with IGF-1 (*r *= 0.617, *P* < 0.05) and negatively correlated with the number of X chimaeras (*r *= −0.583, *P* < 0.05); BMISDS had no significant correlation with any of the influencing factors. These data suggest that increased levels of T, T/DHT, GH, and IGF-1 can promote the growth of height.

**Table 5:** Correlation between height, weight, BMI and various indicators in 45,X/46,XY children.

Variable	HtSDS	WtSDS	BMISDS
T after HCG stimulation(nmol/L)	0.636**	0.268	−0.015
DHT after HCG stimulation(pg/ml)	−0.084	0.429	0.443
T:DHT ratio	0.724**	0.093	−0.282
DHEAS(µmol/L)	−0.384	0.201	0.766
INHB (pg/mL)	−0.200	0.311	−0.092
AMH (ng/mL)	0.104	−0.104	−0.162
Basal LH(IU/L)	−0.284	−0.001	0.520
Basal FSH(IU/L)	−0.385	0.063	0.610
Peak LH(IU/L)	−0.252	−0.046	0.396
Peak FSH(IU/L)	−0.351	−0.078	0.341
IGF-1 (ng/ml)	0.067	0.617*	0.907
IGF-3 (µg/ml)	−0.089	0.443	0.791
Peak GH(µg/L)	0.827*	0.602	−0.235
Gonadal pathology with female gonads tissue	−0.663*	−0.057	0.298
degree of 45,X mosaicism	−0.029	−0.583*	−0.329
degree of 46,XY mosaicism	−0.533*	0.015	−0.400

IGF-1,insulin-like growth factor-1; IFGBP-3, insulin-like growth factor-binding protein-3; GH, growth hormone; The values in the table are correlation coefficients; two variables are compared, *P < 0.05, **P < 0.01.

**Table 6:** Regression analysis of HtSDS, WtSDS and various indicators in 45,X/46,XY children.

Dependent variable	Independent variable	Constant	*B*	SE	*β*	*t*	*P*
HtSDS	T after HCG stimulation	−2.915	0.24	0.073	0.636	3.3	0.005
	Peak GH	−5.45	0.365	0.124	0.827	2.943	0.042
	Gonadal pathology with female gonads tissue	−0.607	−0.61	0.208	−0.663	−2.933	0.014
	Chimaera XY number	−0.907	−0.028	0.01	−0.533	−2.674	0.015
WtSDS	IGF-1	−2.915	0.24	0.073	0.636	3.3	0.005
	degree of 45,X mosaicism	−0.907	−0.028	0.01	−0.533	−2.674	0.015

**Figure 4:**
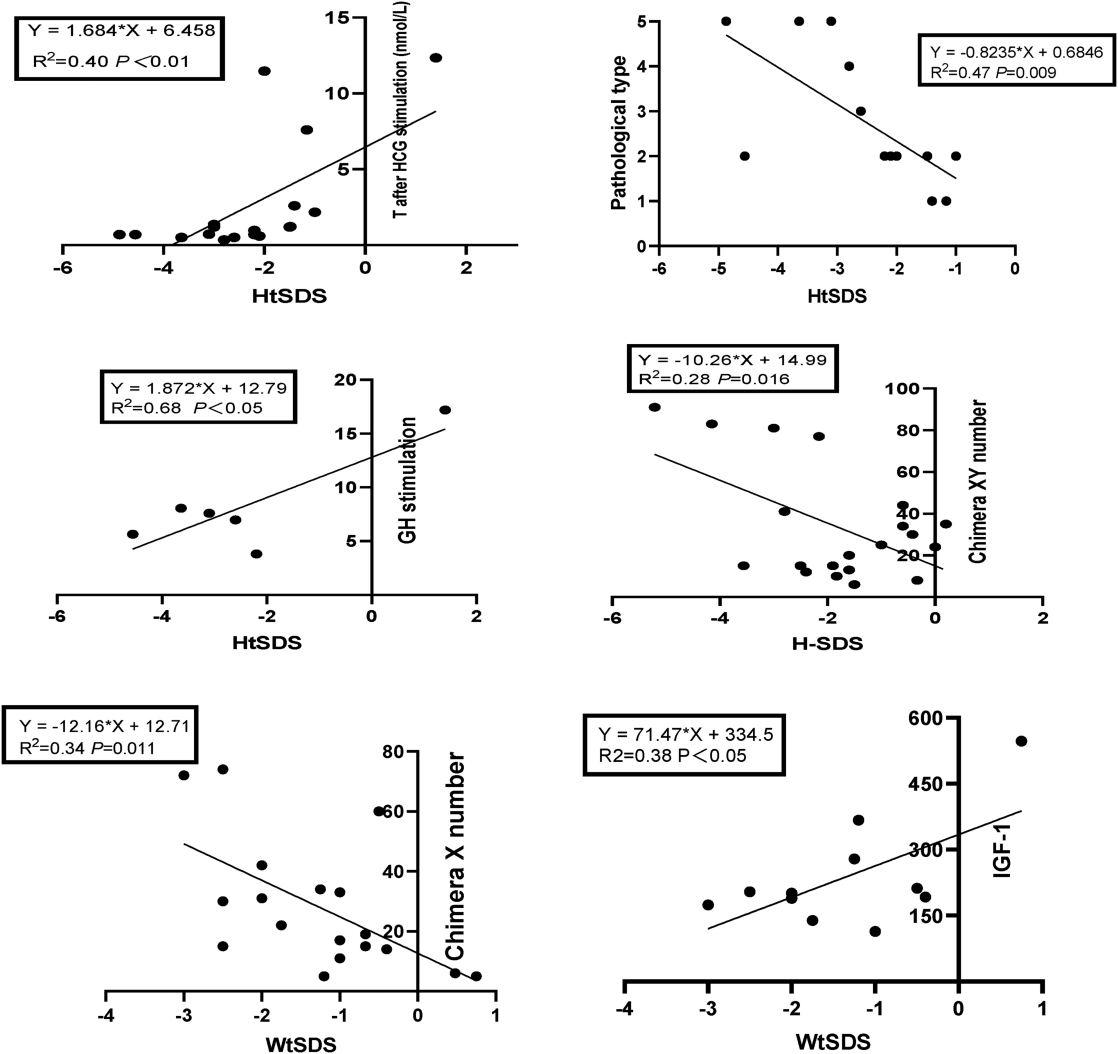
Regression equation of height SDS, weight SDS and related influencing factors in 45,X/46,XY patients.

Taking HtSDS and WtSDS as dependent variables and the related influencing factors as independent variables, multiple stepwise regression analysis was carried out (Figure 4). The results showed that the height of 45,X/46,XY children is affected by many factors. Under the control of other factors, T levels can affect the growth of height, and height increases as T levels increase. GH can affect height. Among 38 children, a total of 7 children received growth hormone stimulation tests. The higher the result of the stimulation test was, the taller the child. The pathological types were classified according to the pathological results, and twenty-nine cases underwent bilateral gonad biopsies. The results showed that children with testicular tissue in the bilateral biopsy results were the tallest, followed by the mixed type. The number of XY chimaeras was inversely proportional to height. As the number of XY cells in the serum increased, the height decreased, indicating that the degree of XY chimaerism in the blood of 45,X/46,XY children cannot determine their level of masculinization.

## Discussion

45,X/46,XY mosaicism is the most common type of sex chromosomal abnormality DSD, with an incidence of 1.0/10,000 (6). The mechanism of the abnormality may be that the Y chromosome lags behind and does not segregate when the fertilized egg undergoes mitosis (7). Although some studies have shown that the prenatal sex chromosomes are 45,X/46,XY, 95% of cases have normal male sex organs after birth (8). In fact, the phenotype of the external genitalia of the disease is broad, including the appearance of female genitalia or mild clitoromegaly through ambiguous genitalia to hypospadias or a normal penis (9). In our study, a total of 38 children with sex chromosome karyotypes of 45,X/46,XY were included as research subjects. These patients were divided into a male phenotype group (20 cases) and a female phenotype group (18 cases) according to their external genital phenotype. The EMS of patients raised as female was 1 (0–3) [median (range)], and that of males was 7.63 (3–11) [median (range)]. EMS < 3 points indicates a female phenotype, and EMS > 3 points indicates a male phenotype.

In addition to external genital abnormalities, such children will also have short stature problems. At present, there are few reports on the growth of 45,X/46,XY individuals during childhood. A previous study (6) analyzed the height of 32 cases of 45,X/46,XY and found that patients with 45,X/46,XY might have normal heights until 2 years old, but growth decelerations after 2 years of age were common. In this study, we analysed the growth patterns of these children, and the results showed that these children have specific growth patterns. Their HtSDS was in the normal range from 0 to 2 years old, and after that, their age was negatively correlated with HtSDS since after 2 years of age, their growth began to slow. The probability of short stature in females (67%) was higher than that in males (45%). In this study, 7 children underwent growth hormone stimulation testing; 6 had short stature (HtSDS < 2 SD) and they were predominantly female (83%). The results of the two GH drug (clonidine, arginine) provocation tests in these 6 children were all less than 10 µg/L, suggesting the existence of growth hormone deficiency. The detection of IGF-1 and IGFBP-3 was conducted in 13 children, and 4 had lower IGF-1 than normal children, and the 6 children with bone age records all had bone age lag >2 SD. This shows that children with short stature 45,X/46,XY have growth hormone deficiency, a low bone age, and low levels of growth factors.

A previous report of 10 patients placed on growth hormone (GH) therapy found that although the HtSDS of the GH-treated patients was significantly higher than their mean HtSDS before GH treatment (*P *= 0.013), it was not significantly different from the HtSDS of the untreated group (7). This may be related to the short treatment time and the lack of the SHOX gene on chromosome X. In another study (2), in their male group, fourteen of 18 males had external masculinization scores consistent with normal virilization. Ten of 11 male patients experienced spontaneous puberty, gonadal function in most 45,X/46,XY males, even those with genital ambiguity, seems sufficient for spontaneous puberty. While 4 female patients in our study entered puberty but no development of secondary sexual characteristics, this further indicates that in such children, some testicular function is preserved in the male group, while the gonadal damage is more severe in the female group.

In this study, the WtSDS of 45,X/46,XY children in different age groups were all within the normal range but below the median, and there was no significant difference between males and females. The BMI of the different age groups was different, and it was positively correlated with age. The older the child, the higher the BMI, and the BMISDS was in the normal range with no manifestation of obesity. The BMISDS of females was higher than that of males. Due to the calculation method of BMI, this may be caused by males being taller than females.

AMH and INHB are markers suggesting the existence of Sertoli cells, and AMH and INHB play an important role in gonadal development and sex differentiation. Assessment of AMH and INHB helps determine the testicular presence and function. In patients with bilateral cryptorchidism, undetectable serum AMH and INHB suggest testicular tissue loss. The levels of AMH and INHB in the male phenotype group were significantly higher than those in the female phenotype group (*P *< 0.05). Compared with the reference range reported in the literature (10), the AMH of the male group was in the normal range, and that of the female group was lower than the normal range, suggesting that AMH and INHB are very important for evaluating testicular function and determining the sex of rearing.

All 38 children with 45,X/46,XY underwent an HCG stimulation test. The T level after the HCG challenge test was significantly higher in the male phenotype group than in the female phenotype group (*P* < 0.001). The male group had higher T:DHT ratio values than the female group (*P* < 0.001). This further shows that the testis function of the male group is better than that of the female group and also that there is insufficient function of 5α-reductase to convert T to DHT, indicating that the increase in the T:DHT ratio is not specific to 5α-reductase deficiency.

Thirty-seven cases of 45,X/46,XY children had a GnRH stimulation test, these data showed that the basal LH and FSH levels and the peak LH and FSH levels of the female group were higher than those of the male group. At the same time, they were significantly higher than that of normal children, and there was a phenomenon of hypergonadotropic hypogonadism in 45,X/46,XY children. Combined with the significant reduction of AMH and INHB in the female group, this further confirmed that the females had obvious hypogonadism. In the female group, 13 cases underwent bilateral gonad biopsy, for which the pathological results were2 cases of male gonad tissue on both sides, 1 case of bilateral female gonad tissue, 6 cases of mixed male and female gonad tissue, and 1 case of bilateral streak gonads (Table 7). These data all suggest that the female group has different types of hypogonadism and an increased tumour risk. In terms of gonad pathology and gonadectomy, In a previous study (11), they considered in girls, tumor risk is limited but gonads are not functional, making gonadectomy the most reasonable option, but in our study, in the female group, 9 children underwent bilateral dysplastic gonadectomy, and in the male group, 6 children underwent unilateral gonadectomy, not only because of the tumor, the extremely poor development of the gonads is also the reason for the gonadectomy, so evaluation for gonadectomy is necessary in both males and females.

**Table 7:** Histological findings in patients who underwent surgical exploration and/or gonadectomy.

Patient no.	Sex	Surgery	Histology	
			Left gonad	Right gonad
2	M	at 4 years B(right) O(left)		Streak gonad, no germ cells
3	M	at 1 years G(left)	epididymis and fallopian tube-like tissue	
4	M	at 23 months B(bilateral) O(bilateral)	Seminiferous tubule	Epididymis tissue, no seminiferous tubules
5	M	at 14 months B(bilateral) G(right)	Fibrous tissue, smooth muscle, lining columnar epithelium, oviduct-like structures	Testicular tissue
7	M	at 15 months B(bilateral) O(bilateral)	Seminiferous tubules, Sertoli cells, germ cells, and stromal cells	Seminiferous tubules, Sertoli cells, germ cells, and mesenchymal cells
8	M	at 13 months B(bilateral)	Seminiferous tubules, Sertoli cells and germ cells	Seminiferous tubules, Sertoli cells and germ cells
9	M	at 3.5 years B(bilateral)	Seminiferous tubule	Few seminiferous tubules
11	M	at 21 months B(bilateral) O(bilateral)	Streak gonad, no germ cells	Seminiferous tubule
12	M	at 13 months B(bilateral) O(bilateral)	Few seminiferous tubules	Few seminiferous tubules
13	M	at 17 months B(bilateral) O(left) at19 months G(right)	Seminiferous tubule	Fibrovascular connective tissue
14	M	at 18 months B(bilateral) at 2.5 years 0 (left)	Seminiferous tubules	Few seminiferous tubules
15	M	at 27 months B(bilateral) G(left)	Fallopian tube and vas deferens structure	Few seminiferous tubules
16	M	at 15 months B(bilateral)	Streak gonad with some seminiferous tubules	Streak gonad with some seminiferous tubules
18	M	at 13 months B(bilateral) at 16 months G(left)	A small amount of ovarian stroma, no follicles and seminiferous tubules	Seminiferous tubules
19	M	at 22 months B(bilateral) at 4 years G(right)	Seminiferous tubule, epididymis	Streak gonad with a few tubules
20	M	at 16 months B(bilateral) O(left)	Few seminiferous tubules	Ovarian mesenchymal tissue
22	F	at 8 years B(bilateral)	Streak gonad, no germ cells	Streak gonad, no germ cells
23	F	at 13 years B(bilateral) G(bilateral)	Streak gonad with small glandular duct	Streak gonad with small glandular ducts and more calcifications
26	F	at 1.5 years B(bilateral)	Seminiferous tubules	Seminiferous tubules
27	F	at 15 years B(bilateral)	Seminiferous tubules	Streak gonad, no germ cells
28	F	at 19 minths B(bilateral)	A small amount of ovarian tissue is seen, and an immature follicle can be seen	Few seminiferous tubules
29	F	at 11 years B(bilateral) G(bilateral)	Streak gonad with few cavity structures	Epithelial nest structure
31	F	at 4.5 yr B(bilateral) G(bilateral)	Fibrovascular fatty nodules, Fallopian tube structure, epididymis and vas deferens structure	Fibrovascular fatty nodules, oviduct and vas deferens structures
32	F	at 55 months B(bilateral) G(bilateral)	Several glandular structures were seen in the ovarian-like stroma, but no obvious follicle tissue was seen	Fallopian tube structure and ovarian-like stroma, surrounded by lumen structure and a little vas deferens
33	F	at 68 months B(bilateral) G(bilateral)	Tumors of germ cell origin	Streak gonad, no germ cells
35	F	at 10 years B(bilateral) G(bilateral)	Gonadoblastoma	Slight ovarian-like stroma, vas deferens
36	F	at 16 years B(bilateral) G(bilateral)	A small amount of ovarian stroma and fallopian tubes	A small amount of ovarian stroma and fallopian tubes
37	F	at 13 years B(bilateral) G(bilateral)	Streak gonad with fallopian tube tissue	Gonadoblastoma
38	F	at 10 years B(bilateral) at 11 years G(bilateral)	Streak gonad, few glands	Streak gonad, no germ cells

Patient numbers are the same as in Tables 1, 2. M, Male; F, female; B, biopsy; G, gonadectomy; O, orchiopexy.

In our study, a correlation analysis of height, weight, BMI and related factors was carried out. In this type of disease, the height of the children is related to testosterone, growth factors, and pathological types. T can directly stimulate the secretion of GH by interacting with the androgen receptor (AR) and oestrogen receptor (ER) located in the hypothalamus and pituitary and can also be converted to oestrogen through peripheral and central aromatization, indirectly affecting circulating IGF-1 (12). In addition, the perinatal surge of T can imprint the GH/IGF-1 axis, regulating pubertal GH secretion, body weight, and longitudinal bone growth (13). Our data indicate that height in 45,X/46,XY children is positively correlated with T.

The pituitary secretes pulsatile growth hormone (GH) and it acts directly or indirectly on peripheral tissues by stimulating the synthesis and secretion of IGF-1 (14). IGF-1 induces chondrocyte proliferation and endochondral ossification, leading to linear bone growth upon stimulation with GH (15). A total of 7 children underwent a growth hormone stimulation test, and the growth hormone stimulation test results were proportional to the height of the child.

The children were further classified according to their pathological results. Among them, 29 cases underwent bilateral gonad biopsies. The pathological types were testicular tissue, mixed ovarian and testis tissue, ovarian tissue, no gonad tissue, and germ cell tumour. We compared the pathological results with the T level. There was a correlation between the pathological types and height. The pathological type with the tallest children was testicular tissue with the highest T level, followed by the ovo-testis mixed type, and the shortest were those with a germ cell tumour.

IGF-1 binds to its receptor and plays an important role in growth and development. IGF-1 can bind to six types of IGFBP in the blood circulation to regulate the activity of IGF-1, of which the most abundant is IGFBP-3, which accounts for 80%–95% (16). Recent studies have shown that intrauterine IGF-1 levels can affect the birth weight of infants (17). It has been shown that children with this type of disease can be treated with GH to alter their GH-IGF-1 levels, thereby altering their growth.

## Conclusions

Patients with 45,X/46,XY mosaicism have specific growth patterns. Their HtSDS was in the normal range during 0–2 years of age, and then they began to show short stature after 2 years of age. The probability of short stature in females was higher than that in males. Short stature patients had growth hormone deficiency, retardation of bone age, and low IGF. Their WtSDS were all in the normal range but below the median. BMISDS was in the normal range, and there was no evidence of obesity.

The values of INHB, AMH, T (after HCG stimulation), and the T:DHT ratio in the male group were significantly higher than those in the female group, and the values of LH, FSH, peak LH and peak FSH in the female group were significantly higher than those in the male group. These data suggested that the gonads in the male group retained a certain androgen secretion function. The female group had impaired gonadal function, manifesting as hypergonadotropic hypogonadism. The hormone levels in the two groups can help us better understand this type of DSD disease, provide a basis for sex selection, and assist in the development of a personalized therapeutic schedule.

## Data Availability

The original contributions presented in the study are included in the article/**Supplementary Material**, further inquiries can be directed to the corresponding author/s.
