# Supplementary material for: Clinical and hormonal characteristics and growth data of 45,X/46,XY mosaicism in 38 Chinese patients
Source: Front Pediatr. 2023 Apr 19;11:1135776. doi: 10.3389/fped.2023.1135776 (PMC10154695; doi:10.3389/fped.2023.1135776)
Supplement: Supplementary file 1 [file Table1.docx]

|  | **Clonidine stimulation test** | | | | | | **Arginine stimulation test** | | | | | |
| --- | --- | --- | --- | --- | --- | --- | --- | --- | --- | --- | --- | --- |
| no. | 0min | 30min | 60min | 90min | 120min | Peak | 0min | 30min | 60min | 90min | 120min | Peak |
| 23 | 1.62 | 0.75 | 6.97 | 4.91 | 3.84 | 6.97 | 0.21 | 1.04 | 3.6 | 5.22 | 2.08 | 3.6 |
| 24 | 1.22 | 0.87 | 2.11 | 3.81 | 2.43 | 3.81 | 0.54 | 1.57 | 9.8 | 8.5 | 6.43 | 9.8 |
| 25 | 0.28 | 4.2 | 17.9 | 16 | 11.7 | 17.19 | 3.63 | 7.12 | 8.32 | 5.19 | 1.74 | 8.32 |
| 29 | 0.81 | 0.17 | 6.56 | 5.64 | 3.54 | 5.64 | 0.14 | 3 | 2.81 | 0.63 | 0.23 | 3 |
| 10 | 0.59 | 0.79 | 9.94 | 7.5 | 2.51 | 9.94 | 0.28 | 7.35 | 9.22 | 2.85 | 0.83 | 9.22 |
| 33 | 8.03 | 2.93 | 8.05 | 6.56 | 1.83 | 8.05 | 0.4 | 1.3 | 2.93 | 0.88 | 0.33 | 2.93 |
| 35 | 0.22 | 0.41 | 7.59 | 7.21 | 2.77 | 7.59 | 0.23 | 0.45 | 0.23 | 0.19 | 0.19 | 0.45 |
